# Adaptive Skills and Somatization in Children with Epilepsy

**DOI:** 10.1155/2014/856735

**Published:** 2014-01-27

**Authors:** Nichole Wicker Villarreal, Cynthia A. Riccio, Morris J. Cohen, Yong Park

**Affiliations:** ^1^Texas A&M University, College Station, TX 77843-4225, USA; ^2^Department of Educational Psychology, Texas A&M University, College Station, TX 77843-4225, USA; ^3^Children's Hospital of Georgia, BT-2601, 1446 Harper Street, Augusta, GA 30912, USA

## Abstract

*Objective*. Children with epilepsy are at risk for less than optimum long-term outcomes. The type and severity of their epilepsy may contribute to educational, psychological, and social outcomes. The objective of this study was to determine the relation between somatization and adaptive skills based on seizure type that could impact on those outcomes. *Methods*. This study examined adaptive functioning and somatization in 87 children with epilepsy using archival data from a tertiary care facility. *Results*. No significant differences in adaptive skills emerged between groups of children diagnosed with complex partial (CP) as compared to CP-secondary generalized (SG) seizures; however, deficits in adaptive behavior were found for both groups. The number of medications, possibly reflecting the severity of the epilepsy, was highly correlated to adaptive function. *Conclusions*. Identification of deficits in adaptive behavior may represent an opportunity for tailored prevention and intervention programming for children with epilepsy. Addressing functional deficits may lead to improved outcomes for these children.

## 1. Introduction

Among chronic illnesses, epilepsy is the most common neurological condition in childhood [1–3]. Childhood epilepsy is associated with multiple causes including neurological deficit present at birth, cerebrovascular disease, trauma, tumor, or infection. Often the cause is unknown or designated as idiopathic. Not all children experience the same type of seizure and potential effects on educational and psychological outcome may differ by seizure type or etiology. Epilepsy, regardless of seizure type, has been increasingly recognized as a risk factor for potential negative outcomes for children and their families, from childhood into adulthood [3–5]. Specifically, long-term studies have shown that the social and psychological outcomes of adults with childhood onset of epilepsy are poorer than for typical peers with regard to educational level, employment, independent functioning, and socioeconomic status [6–9]. Although it is not uncommon for epilepsy to be associated with lower cognitive functioning, the negative outcomes are not specific to those with impaired cognition. Studies conducted on young adults with normal cognitive ability and a history of childhood epilepsy also found lower than expected levels of educational attainment, greater unemployment, lower socioeconomic status, and lower marriage rates [6, 7, 10, 11].

Studies have highlighted the relationship between seizure variables (e.g., age at onset, time since diagnosis, and medications), psychological well-being, and independent functioning [8, 12–16]. A number of studies have concluded that epilepsy-related factors are not strong predictors of psychopathology in childhood [17, 18]. In contrast, there are indications that demonstration of adaptive behavior may be associated with seizure severity and control [2, 5, 19, 20]. Adaptive behavior encompasses a range of behaviors that are considered essential for everyday functioning, including daily living skills, social competence, functional communication, leadership, and adaptability.

At best, Sillanpää and Cross [5] found that although most individuals with epilepsy were able to function independently, the risk of deficits in independent living was greatest in those with complicated epilepsy. Clary et al. [21] found children and adolescents to evidence significant deficits in both daily living skills and functional communication. When children with normal cognitive ability were considered separately, results still indicated significant deficits across adaptive areas, including socialization, communication, and daily living skills [22]. Other studies found that children with epilepsy evidenced significant adaptive behavior deficits not explained by cognitive deficits [23, 24]. Of the adaptive skills, social and communication skills were found to predict later school performance in children with early-onset epilepsy [25].

In addition to potentially impaired adaptive skills, associated with health and medical concerns, children with epilepsy may exhibit a higher than expected level of somatic complaints, potentially indicative of somatization. From a psychological perspective, somatization is best described as the presentation of a psychological problem (e.g., anxiety or depression) as a physical complaint. Specific behaviors (e.g., complaints of headache or stomach ache and taking medication) in an otherwise healthy individual oftentimes are interpreted in this manner. For children with epilepsy, these behaviors may reflect the medical condition as opposed to (or in addition to) an underlying psychological problem. Somatic complaints have been found to be more common among children with lower cognitive abilities [26]; thus, the cognitive ability differences in a sample could affect results. Notably, Clary et al. [21] did not find elevated mean somatization scores in their sample but reported that 17% of the participants did reach clinically significant levels. Little research has examined the extent to which children and adolescents with epilepsy exhibit somatic complaints or the extent to which somatization is related to adaptive skill development.

Although epilepsy is by far one of the most studied and explored chronic illnesses, research has not examined the relationship between somatization and general adaptive functioning to the same extent that risk for psychopathology has been explored. Existing data suggest that the presence of epilepsy increases the likelihood of somatic complaints and low adaptive skills. The likelihood of these problems developing is increased by poor cognitive functioning and behavioral problems, potentially contributing to social skill deficits [12, 13, 22, 27, 28]. The connection between cognition and behavioral functioning is well established. In contrast, the association between adaptive skills and somatic complaints is not well established. A clearer understanding of levels of adaptive functioning for children with epilepsy and the relationship between adaptive skills and somatic complaints could provide a better framework for working with these children. Moreover, psychosocial and adaptive functioning are not only predictive of long-term outcome, but also have been found to be related to quality of life in children and adolescents with epilepsy [21, 29]. The purpose of this study was to add to the growing literature on the relation of aspects of adaptive functioning, levels of somatization, and epilepsy-related factors. It was hypothesized that there would be differences in level of somatization and adaptive behavior for children with complex partial (CP) seizures as compared to CP-secondary generalized (SG) seizures.

## 2. Materials and Methods

### 2.1. Participants

This is a retrospective study using extant data of children and adolescents with epilepsy who were referred to a tertiary care epilepsy center in the south for neuropsychology evaluation between the years of 1997 and 2010. The 87 participants included those individuals with epilepsy who meet the criteria for inclusion. Children were included if (a) they were between the ages of 4 and 17, (b) they had been administered a version of the Behavior Assessment System for Children BASC [30]/BASC-2 [31] Parent Report, and (c) they had a predominant seizure type of complex partial (CP) or complex partial-secondary generalized (SG) epilepsy at least one year prior to the time of the evaluation. The determination of seizure type was made by a pediatric neurologist based upon the seizure characterization and EEG monitoring. CP and SG are the most common types of seizures seen at this facility and it was of interest to see if secondary generalization would further affect functioning. The following exclusionary criteria were used: (a) children with a diagnosis of epilepsy for less than a year, (b) omission of the BASC/BASC-2 parent report, or (c) cognitive ability below 70. Cognitive ability was restricted in order to control for adaptive behavior deficits that could otherwise be associated with intellectual disability. Teacher reports on the BASC/BASC-2 were available on some but not all of the participants. Requiring the teacher form would have significantly reduced the sample size; however, results of the teacher form are reported.

Participants (*N* = 87) were predominantly male (59.77%) and Caucasian (72.41%). The participants had a mean age of 10.65 (SD = 2.89). The participants' cognitive ability ranged from 70 to 115, with a mean IQ of 86.26 (SD = 10.89). Of the participants, 64 (73.56%) had a diagnosis of CP and 23 (26.44%) had a diagnosis of SG. The two groups did not differ in age at the time of assessment or full scale IQ. Seizure variables of interest included age of onset, duration, and number of medications. In particular, number of medications was used as an indicator of severity; number of medications for both groups ranged from 0 to 3. Most frequent antiepileptic medications included one or more of the following: oxcarbazepine (36% of participants), levetiracetam (28%), carbamazepine (22%), lamotrigine (19%), or valproate (16%). No between group differences were found for age at onset, duration, or number of medications. Similarly, chi-square analyses indicated that groups did not differ significantly in sex, race/ethnicity, or reported family income.  Table 1 provides the demographic data by seizure type.

### 2.2. Procedures

This study was a retrospective research project using an existing data base. The primary measures of interest were parent-/guardian- and teacher-rating scales of the child's behaviors, as well as information pertaining to the child's medical history. Approval for the study was obtained from the Medical College of Georgia Institutional Review Board (IRB) and then by the Texas A&M University IRB to use the extant data base. Because the data for this study were archival, collection and coding systems were already established. Demographic information including age, race/ethnicity, gender, income, and type of epilepsy, as well as dependent and independent variables used in this study, was provided in the database. Only those cases meeting the inclusion and exclusion criteria were retained. Data were collected from comprehensive neuropsychological reports completed with appropriate parental and child consent at the tertiary care epilepsy center. The dependent variables that were examined in this study were the adaptive skills and level of somatization as reported by parents and teachers, as well as specific epilepsy-related variables.

### 2.3. Measures

*Wechsler Intelligence Scale for Children* [32, 33]. The Wechsler scales are commonly used measures of ability for children and adults. Because of the retrospective nature of the study, children had different editions of the Wechsler scales. Each of the participants in this study was given the WISC-III or WISC-IV, as a measure of cognitive ability. For the purposes of this study, the results of the WISC-III and WISC-IV are included for descriptive purposes with consideration of the full scale score only. The correlation of the WISC-IV with the previous version of the WISC-III is high (*r* = 0.89). For this sample, 51 (68.62%) of the participants were administered the WISC-IV.

*Behavior Assessment System for Children [30, 31]*. The BASC/BASC-2 is used to evaluate the behavior and self-perceptions of children and adults aged 2 through 25 years of age. The BASC/BASC-2 includes forms for preschool, child, and adolescent. The BASC/BASC-2 has been well recognized as an appropriate instrument for the evaluation of behavior in children and adolescents [34]. There are specific forms of the BASC/BASC-2 based on the age of the individual, as well as the relationship of the rater to the individual (e.g., parent, teacher, and self). The interpretation of the BASC/BASC-2 scales and subscales is based on T-scores. T-scores from 60 through 69 on the clinical scales and 31 through 40 on the adaptive scales are considered at risk. T-scores of 70 and above on clinical scales and 30 and below on adaptive scales are considered clinically significant. The BASC/BASC-2 includes two validity scales such that records likely to be invalid for reasons such as carelessness, inattentiveness, or cognitive limitations could be eliminated. Additionally, the BASC/BASC-2 demonstrates good convergent validity with other measures [34, 35].

For the purposes of this study, the variables of interest included the adaptive skills subscale scores, as well as the somatization subscale from the internalizing composite. The adaptive skills composite includes the following subscales: adaptability, social skills, functional communication, leadership, learning problems (teacher only), study skills (teacher only), and activities of daily living (parent only) depending on the form and version used. The coefficient alpha across forms and version is greater than .80 except for the activities of daily living subscale (.72–.76).

The Somatization subscale assesses the tendency of the child or adolescent to be report physical complaints or seek medical intervention. It is composed of items that cover topics including doctors' visits and physical ailments (e.g., visits school nurse, gets sick, has headaches, is afraid of getting sick, makes frequent visits to the doctor, and so on). The reliability coefficients for the somatization subscale for children aged 6 to 18 range from .79 to .84. Convergent validity for this subscale has been demonstrated as well [30, 31].

For this study, both child and adolescent forms were administered depending upon the child's age. It should be noted that not all forms included all subscales. Further, using retrospective data meant that some participants had the original BASC and others had the BASC-2. Although correlations between the composite scores for the two versions are good (.89–.98), the functional communication subscale was not a component of the BASC. As a result of the differences in forms, the number of participants who had specific scales varies based on age, form (parent/teacher), and version.

## 3. Results and Discussion

Preliminary analyses were performed to ensure no violation of the assumptions of normality, linearity, and homoscedasticity on the dependent variables. Across BASC/BASC-2 variables and epilepsy variables (onset, duration, and number of medications), no variables violated assumptions. To compare by seizure type, a two-group design was used. It was hypothesized that the two groups, CP and SG, would differ in level of somatic complaints as well as adaptive skills. Correlational analyses were used to examine relations between variables. Alpha was set at .05 so as to reduce the likelihood of type II error, the greater concern in a small clinical sample. All analyses were conducted using SPSS.

### 3.1. Parent and Teacher Ratings by Seizure Type

It was hypothesized that the children and adolescents in the SG group would demonstrate more difficulties in adaptive functioning and higher somatization scores than those in the CP group. One-way analysis of variance was used to evaluate the differences between epilepsy groups for the BASC/BASC-2 teacher and parent reports. While no significant between group differences were found for parent reports, the groups differed on the teacher ratings of leadership (*P* < .05, partial eta-squared = .08) and study skills (*P* < .05, partial eta-squared = .09). Specifically, as predicted, the SG group mean scores on these subscales were lower than those of the CP group.

Results were also considered by frequency of at risk and clinically significant scores for each of the variables based on z-scores and standard deviation for the BASC/BASC-2 scores (see Table 2). Notably, for both parent and teacher somatization, more than 40% of each group evidenced an elevated score. As suggested by differences in group means, the frequency of at risk and clinically significant scores is much higher for the SG group (45% and 50%) for leadership and study skills as compared to the CP group (26.92% and 24.53%, resp.). Activities of daily living is only a subscale on the parent-child form, with results only available for a small subsample. It is important to note, however, that of those participants in that age bracket, more than 50% for each group evidenced at risk or clinically significant impairment. Notably, the difficulties in these daily living skills are consistent with prior research [5, 21, 22].

### 3.2. Relation of Adaptive Skills with Epilepsy Characteristics

It was hypothesized, for both the parent and teacher reports, that the younger the age at onset of seizures is, the longer the duration of living with the illness and number of medications taken would be correlated with adaptive skills deficits. Correlation (*r*) was used to determine the relation between age of onset, duration, and number of medications with each of the adaptive skills subscales for the entire sample, as well as for somatization (see Table 3). No significant correlations were found with age of onset or duration for either parent or teacher ratings. For the parent ratings, there was a significant correlation between number of medications and adaptability (*P* > .05), Leadership, (*P* < .05), and activities of daily living (*P* < .01), with more somatic complaints and lower adaptive skills associated with more medications needed for seizure control (i.e., severity). For teacher ratings, no significant associations were found with epilepsy variables.

### 3.3. Somatic Complaints and Adaptive Functioning

It was hypothesized that somatization scores would be inversely related to adaptive functioning for both the parent and teacher report, both presumed to reflect severity and impact on functioning. Correlation analysis was conducted to ascertain whether a relationship existed between level of somatic complaints and the adaptive skills subscales as measured by the BASC/BASC-2 parent and teacher reports. The results (see Table 4) indicated that there was relative agreement between parent and teacher ratings of somatization for those children with both respondents information (*n* = 78, *r* = .30, and *P* = .007). The differences between parent and teacher ratings are common [36]. These differences may reflect differences in expectations and contextual demands. Parent-rated somatization was not significantly associated with any of the parent-rated or teacher-rated adaptive skills. In contrast, teacher-rated somatization was significantly related to parent-rated social skills (*P* = .02) and leadership (*P* = .01). Teacher-rated somatization was also significantly correlated with teacher-rated Social Skills (*P* < .05), leadership (*P* = .04), Study Skills (*P* = .007), and functional communication (*P* = .02). In all cases, a higher level of somatic complaints was associated with lower adaptive skills as expected.

## 4. Conclusions

The long term outcome for children with epilepsy tends to be less than optimum [3, 5, 7, 10, 11, 25]. Not only may they encounter difficulty in academic areas, but also the circumstances of their epilepsy (i.e., age of onset, time since diagnosis, and number of medications) may result in frequent somatic complaints and may limit their participation in social or community activities. Deficits in adaptive behavior [5, 22, 25] can affect school performance and overall adjustment. The findings here on the adaptive skills of children with epilepsy in relation to somatization, as well as epilepsy characteristics (i.e., seizure type, age of onset, duration, and number of medications), provide support to and add to the existing knowledge base.

Parents and teachers identified some differing aspects of adaptive behavior to be of concern; these differences likely reflect the differing contextual demands. Surprisingly, neither age of onset nor time since diagnosis was correlated with the somatization or any of the adaptive skills subscales. In contrast, number of medications was significantly correlated with parent-rated, but not teacher-rated adaptive behavior. For all three significant parent subscales, the ratings were lower as the number of medications (i.e., severity) increased.

When exploring the relation between somatization and adaptive skills, results did not indicate parent ratings of somatization to be related to adaptive skills. In contrast, for teacher ratings, there was an inverse relation such that higher levels of somatic complaints were associated with specific adaptive behaviors (study skills, leadership, and social skills). Because the somatization subscale emphasizes physical aches and pains, medications, and medical treatments, these findings suggest that the higher the degree of symptomology the child is experiencing due to their illness, particularly in the school setting, the less likely they are to actively engage and participate in the classroom.

Somatic complaints may be expected in children and adolescents with epilepsy and other chronic illnesses. As a result, somatization scales may sometimes be elevated for children with epilepsy because of factors related to the seizure disorder rather than because of psychopathology. At the same time, experiencing somatic issues related to epilepsy can have an effect on other areas of development, particularly adaptive skills. Identification of adaptive skills deficits may represent an opportunity for tailored prevention and intervention programming for children with epilepsy to improve their overall outcome. For example, if social competence is the area of concern, implementation of social emotional learning program may be appropriate; if peer relations is the area of concern, a social skills program might be considered. As such, more comprehensive assessment of adaptive skills may be appropriate for children with epilepsy regardless of cognitive ability in order to identify adaptive skills in need of support or intervention [22]. Additional research specific to adaptive skills may better explain the underlying mechanisms related to lower acquisition of these skills for children with epilepsy, as well as the extent to which adaptive skills mediate long-term outcome. A larger, prospective study including a more diverse sample would be useful in identifying moderator and mediator variables for epilepsy-related factors and outcome for children with epilepsy.

## Figures and Tables

**Table 1:** Descriptive characteristics by seizure type.

Demographics (*n*, %)	CP (*n* = 64)	SG (*n* = 23)
Sex		
Male	40 (62.50%)	12 (52.17%)
Female	24 (37.50%)	11 (47.83%)
Ethnicity		
Caucasian	48 (75.00%)	15 (65.22%)
African American	11 (17.19%)	7 (11.48%)
Hispanic/other	5 (7.81%)	1 (4.35%)
Annual income		
>$10,000	7 (10.94%)	2 (8.70%)
$10–20,000	11 (17.19%)	6 (26.09%)
$20–30,000	12 (18.75%)	4 (17.39%)
$30–40,000	7 (10.94%)	1 (4.35%)
$40–50,000	5 (7.81%)	3 (13.04%)
>$50,000	22 (34.38%)	7 (11.48%)

	Mean (SD)	Mean (SD)

Age at testing	10.36 (2.89)	11.42 (2.71)
Full scale IQ	86.42 (11.10)	86.48 (10.70)
Age of onset	5.99 (3.46)	6.14 (3.62)
Duration (years since onset)	4.42 (3.77)	5.33 (3.20)
Number of medications	1.72 (0.85)	1.95 (0.77)

CP: complex partial; SG: complex partial-secondary generalized; FSIQ: full scale intelligence quotient.

**Table 2:** Percent impairment for parent and teacher BASC/BASC-2.

	Complex partial (CP)			CP-secondary generalized		
	*n*	% At risk	% Clinically significant	*n*	% At risk	% Clinically significant
Parent variables						
Somatization	64	18.76%	26.56%	23	13.04%	30.43%
Adaptability	55	40.00%	1.82%	20	50.00%	5.00%
Social skills	64	32.81%	12.5%	22	27.27%	4.5%
Leadership	61	29.51%	6.56%	22	45.45%	13.64%
ADL	29	41.38%	10.34%	14	28.57%	28.57%
FuncComm	30	40.00%	3.33%	13	7.69%	38.46%

Teacher variables						
Somatization	57	22.81%	33.33%	21	42.86%	9.52%
Learning problems	55	27.27%	10.91%	20	35.00%	30.00%
Adaptability	49	20.41%	4.08%	18	33.33%	5.56%
Social skills	57	17.86%	1.79%	21	23.80%	4.76%
Leadership	52	25.00%	1.92%	20	45.00%	0%
Study skills	53	24.53%	0%	20	45.00%	5.00%
FuncComm	26	15.38%	3.85%	13	38.46%	15.38%

Note. At risk = *z*-score = ±1; Clinically significant = *z*-score = ±2; FuncComm: functional communication; ADL: activities of daily living.

**Table 3:** Correlations for parent/teacher report of adaptive subscales with epilepsy variables (rho) and parent-/teacher-rated somatization (Pearson's *r*).

	Onset	Duration	Number medications	
	*r*	*r*	*r*	
Parent variables				
Somatization (*n* = 87)	−.03	−.16	.13	
Adaptability (*n* = 75)	.05	−.13	−.28*	
Social skills (*n* = 87)	.01	−.16	−.15	
Leadership (*n* = 83)	−.01	−.20	−.26*	
ADL (*n* = 43)	.23	−.25	−.41**	
FuncComm (*n* = 43)	−.01	−.22	−.22	

Teacher variables				
Somatization (*n* = 78)	−.04	−.08	.19	
Adaptability (*n* = 67)	−.08	−.03	.12	
Social skills (*n* = 78)	.03	−.07	−.11	
Leadership (*n* = 72)	.02	−.02	−.01	
Study skills (*n* = 73)	.01	−.08	−.15	
FuncComm (*n* = 39)	.31	−.31	.13	

Notes. FuncComm: functional communication; ADL: activities of daily living.

*P < .05.

**P < .01.

**Table 4:** Relation between somatization and adaptive skills.

	Parent somatization		Teacher somatization	
	*r*	*n*	*r*	*n*
Parent variables				
Somatization	1.00	87	.30**	78
Adaptability	−.21	75	−.06	68
Social skills	−.04	86	−.27*	77
Leadership	−.07	83	−.30**	74
ADL	−.17	43	−.28	40
FuncComm	−.20	43	−.02	39

Teacher variables				
Somatization	.30∗∗	74	1.00	74
Adaptability	.12	63	−.11	63
Social skills	−.10	74	−.23*	74
Leadership	−.10	68	−.25*	68
Study skills	.01	73	−.31**	69
FuncComm	−.06	39	−.37*	35

Notes. FuncComm: functional communication; ADL: activities of daily living.

*P < .05.

**P < .01.
